# Habitat-structured fungal mycobiomes at the water–gill interface of farmed red tilapia in Central Thailand: An internal transcribed spacer rRNA amplicon sequencing study

**DOI:** 10.14202/vetworld.2026.1196-1214

**Published:** 2026-03-23

**Authors:** Geraldine Dayrit, Mahmoud Mabrok, Sage Chaiyapechara, Channarong Rodkhum

**Affiliations:** 1 Department of Medical Microbiology, College of Public Health, University of the Philippines Manila, Manila, Philippines; 2 Center of Excellence in Fish Infectious Diseases, Department of Veterinary Microbiology, Faculty of Veterinary Science, Chulalongkorn University, Bangkok, Thailand; 3 Department of Aquatic Animal Medicine, Faculty of Veterinary Medicine, Suez Canal University, Ismailia 41522, Egypt; 4 Aquatic Molecular Genetics and Biotechnology Laboratory, National Center for Genetic Engineering and Biotechnology, National Science and Technology Development Agency, 113 Paholyothin Rd., Klong 1, Klong Luang, Pathumthani 12120, Thailand

**Keywords:** aquaculture microbiome, fish gills, fungal diversity, fungal mycobiome, ITS rRNA sequencing, One Health, red tilapia, water microbiome

## Abstract

**Background and Aim:**

Tilapia aquaculture is rapidly expanding across Southeast Asia and plays a critical role in regional food security. While bacterial microbiomes of farmed fish have been widely investigated, the fungal component of aquatic microbial communities remains poorly characterized, particularly at the biologically important interface between rearing water and fish gills. Fungi may influence fish health, environmental microbial ecology, and occupational exposure risks within aquaculture systems. This study aimed to characterize fungal mycobiomes associated with rearing water and gills of clinically healthy red tilapia (*Oreochromis* spp. hybrids) cultured in Central Thailand using internal transcribed spacer (ITS) rRNA amplicon sequencing and to determine how habitat type, farming system, and environmental variables shape fungal community structure.

**Materials and Methods:**

Samples were collected from ten tilapia farms located in five provinces of Central Thailand, representing two aquaculture systems: open river cages and closed earthen ponds. A total of 27 rearing water samples and 30 composite gill samples were analyzed. Fungal DNA was extracted and the ITS1 region was amplified and sequenced using the Illumina MiSeq platform. Sequence processing and amplicon sequence variant inference were performed in QIIME2 using the DADA2 pipeline. Alpha diversity indices and beta diversity analyses were used to evaluate community structure, while multivariate statistical approaches assessed the influence of habitat type, geographic location, farming style, and physicochemical water parameters.

**Results:**

Fungal communities displayed considerable taxonomic diversity and differed significantly between habitats. Rearing water samples exhibited significantly higher alpha diversity than gill-associated communities. Dominant genera included *Cladosporium*, *Candida*, *Aspergillus*, *Fusarium*, and *Rhodotorula*. Gill communities were relatively enriched in *Candida* and *Fusarium*, whereas rearing water contained higher abundances of *Cladosporium* and *Rhodotorula*. Beta diversity analyses demonstrated significant effects of sampling source, province, and farming system on fungal community composition. Environmental parameters such as pH, nitrate concentration, and ionic strength were associated with variations in fungal diversity, particularly in rearing water. Several detected genera included taxa with known opportunistic pathogenic potential for fish and humans.

**Conclusion:**

This study provides the first ITS-based baseline characterization of fungal mycobiomes associated with red tilapia aquaculture systems in Central Thailand. Distinct fungal assemblages occur at the water–gill interface, with environmental conditions and aquaculture practices influencing community composition. The presence of opportunistic fungal genera highlights the importance of incorporating fungal community monitoring into aquaculture biosecurity and One Health surveillance frameworks to support sustainable fish production, environmental health, and occupational safety.

## INTRODUCTION

Tilapia aquaculture is a key pillar of global food security, particularly in low- and middle-income countries, where it plays a critical role in supporting household nutrition, sustaining livelihoods, and contributing to economic development across the aquaculture value chain [1–4]. Red tilapia hybrids are widely farmed in Central Thailand and Southeast Asia because of their rapid growth, tolerance of variable water quality, and strong market demand [4–6]. As production intensified, disease outbreaks emerged as major constraints on the profitability and long-term sustainability of tilapia aquaculture.

To date, most research and management efforts have focused on bacterial and viral pathogens and on optimizing water quality parameters [7–12]. Fungal infections in fish are increasingly recognized as an emerging challenge for aquaculture performance and food safety, yet there is a scarcity of baseline information on fungal communities in tropical tilapia systems [13, 14].

Microbiome research has substantially advanced our understanding of how host-associated microbial communities in aquatic vertebrates contribute to health and disease [15–18]. Investigations of bacterial assemblages on fish skin, gills, and gut consistently demonstrate that the mucosal microbiota differs markedly from that in surrounding water and is sensitive to environmental gradients, husbandry practices, and co-occurring stressors [7, 16, 19–21].

In contrast, the fungal component of these microbial communities has received limited attention, particularly within warm-water freshwater aquaculture systems [13, 22–25]. Existing studies have documented fungal diversity in recirculating and biofloc systems, pond soils, and riverine environments and have highlighted the occurrence of genera with recognized pathogenic or opportunistic potential for both fish and humans [13, 26–29].

Despite increasing recognition of the importance of microbial communities in aquaculture systems, most microbiome studies in fish have predominantly focused on bacterial assemblages associated with the gut, skin, and surrounding aquatic environment. Consequently, the fungal component of the microbiome, commonly referred to as the mycobiome, remains relatively underexplored in freshwater aquaculture systems, particularly in tropical regions. Existing investigations have mainly documented fungal diversity in environmental matrixes such as pond soils, biofloc systems, and recirculating aquaculture facilities [13, 22–25]. However, comparatively little is known about how fungal communities interact with host-associated tissues, especially mucosal surfaces such as fish gills, which serve as critical interfaces between the host and the surrounding aquatic environment.

In tilapia farming systems, the gills are a primary site of microbial colonization due to their direct, continuous exposure to rearing water. Although several studies have demonstrated that bacterial communities differ markedly between host tissues and surrounding water, similar comprehensive assessments of fungal assemblages across these habitats remain scarce [7, 16, 19–21]. Furthermore, aquaculture production systems such as open river cages and closed earthen ponds present distinct environmental conditions, including differences in hydrology, nutrient dynamics, water exchange, and organic matter accumulation, which may strongly influence microbial community structure [10, 11, 30–32]. Nevertheless, comparative analyses investigating how these production environments shape fungal community composition in both rearing water and fish gills are currently lacking. The absence of such baseline ecological data limits our ability to understand the potential role of fungal communities in fish health, disease emergence, and environmental dissemination within aquaculture ecosystems. Therefore, comprehensive characterization of fungal mycobiomes at the water–gill interface is necessary to establish foundational knowledge for future disease surveillance, environmental monitoring, and One Health–based aquaculture management.

Accordingly, the present study aimed to characterize the fungal mycobiomes associated with rearing water and gills of clinically healthy red tilapia (*Oreochromis* spp. hybrids) cultured in Central Thailand using internal transcribed spacer (ITS) rRNA amplicon sequencing. Specifically, this study sought to (i) compare fungal alpha diversity and phylogenetic diversity (PD) between rearing water and gill-associated communities, (ii) evaluate how sampling habitat, aquaculture production system (open river cages versus closed earthen ponds), and geographic location influence fungal community composition and beta diversity, and (iii) identify dominant fungal taxa, including genera with recognized pathogenic or opportunistic potential for fish and humans. In addition, the study explored the relationships between fungal diversity patterns and physicochemical characteristics of the rearing water to better understand the environmental drivers shaping mycobiome structure. By integrating ecological, environmental, and host-associated perspectives, this work aims to establish the first ITS-based baseline dataset for fungal communities in red tilapia aquaculture systems in Central Thailand and to provide a scientific foundation for future monitoring strategies within a One Health framework.

## MATERIALS AND METHODS

### Ethical approval

All fungal isolation and biosafety procedures were reviewed and approved by the Institutional Biosafety Committee (Approval No. 2431009) of Chulalongkorn University. The Institutional Animal Care and Use Committee of Chulalongkorn University (Approval No. 2431005) reviewed and approved the animal husbandry and experimental procedures involving red tilapia in accordance with the Association of Southeast Asian Nations Good Aquaculture Practices (ASEAN GAqP) for Food Fish (2020). All animal handling, sampling, euthanasia, and biosafety procedures adhered to institutional, national, and international guidelines for the care and use of animals in research, including the ARRIVE 2.0 recommendations, relevant provisions of EU Directive 2010/63/EU, and the World Organization for Animal Health Aquatic Animal Health Code.

Fish were clinically assessed before sampling, and only apparently healthy individuals exhibiting no visible lesions or external signs of disease were included. Euthanasia was conducted via cold stunning by immersion in an ice–water bath at approximately 4°C–6°C until the cessation of opercular movement, in accordance with the American Veterinary Medical Association Guidelines for the Euthanasia of Animals (2020) [33]. Informed consent was obtained from all the participating farm owners prior to sample collection.

### Study area and selection of sites

**Figure 1:**
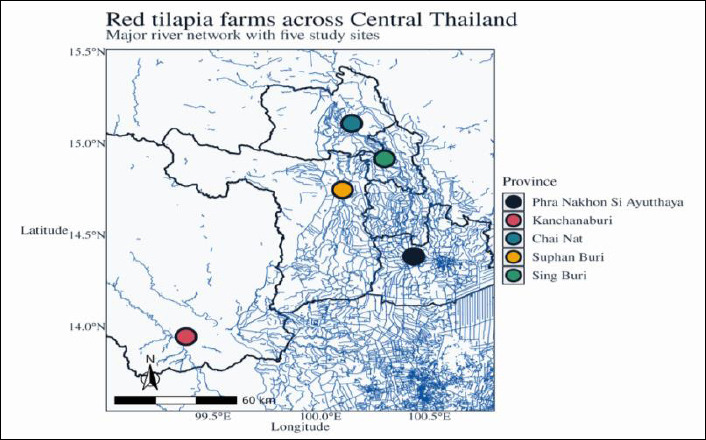
Spatial distribution of red tilapia farms in Central Thailand. Map showing the locations of the five provinces and ten red tilapia farms included in this study. The map illustrates the distribution of open river cages and closed earthen pond farming systems. Symbols distinguish the farming systems (open river cages vs. closed earthen ponds). The base map and river network were generated from publicly available administrative and hydrographic shapefiles. Latitude and longitude are presented in WGS84 coordinates, and the geographic extent of the study area is indicated by a scale bar.

The study was conducted at 10 red tilapia farms in Central Thailand from May to June 2022, corresponding to the late dry to early rainy season, when daytime air temperatures typically range from approximately 30°C to 34°C. Farms were distributed between approximately 13.9–15.1° N and 99.4–100.4° E along major tributaries of the Chao Phraya Basin (Figure 1), including sections of the Chao Phraya, Kwai, Thachin, and Noi Rivers. The spatial distributions of the ten farms across the five provinces are shown in Figure 1.

The Chao Phraya River traverses Chainat, Sing Buri, and Ayutthaya before draining into Thailand’s Gulf. The Kwai River (comprising the Kwai Noi and Kwai Yai branches) is a tributary of the Mae Klong River that flows through Kanchanaburi Province and discharges into the Gulf of Thailand. The Thachin River, a distributary of the Chao Phraya, flows westward through several provinces, including Suphan Buri. The Noi River, which is divided into North and South branches, originates near China, with South China passing through Ayutthaya, an agriculturally and historically significant region in Central Thailand [30, 31].

Water and gill samples were collected from five open river-cage farms and five closed earthen ponds located along these rivers. Farms were selected to capture variation in both provinces (Ayutthaya, Kanchanaburi, Chainat, Suphan Buri, and Sing Buri) and farming systems (open cages versus closed earthen ponds) under real-world production conditions. The inclusion criteria for farms were as follows: (i) a minimum area of 20 m², (ii) operation for at least 3 years, and (iii) the presence of clinically healthy adult red tilapia at the time of sampling. Farm owners reported no recent fungal disease outbreaks during the study period.

Given the exploratory, multisite design and logistical constraints, the sample size was selected to represent this spatial and management gradient rather than being determined through a formal power analysis. Table 1 provides a summary of the study sites, farming systems, and number of samples collected from each source.

**Table 1:** Summary of the study sites, farming styles, and samples collected from each source.

Sampling sites	Major tributary river	Farming style	Water	Gills
Ayutthaya	Noi (South)	Open cages	3	3
		Closed-pond	3	3
Kanchanaburi	Kwai	Open cages	3	3
		Closed-pond	6	6
Chainat	Noi (North)	Open cages	3	3
Suphan Buri	Thachin	Open cages	2	3
		Closed-pond	1	3
Sing Buri	Chao Phraya	Open cages	3	3
		Closed-pond	3	3
Total No.			27	30

### Sample collection

#### Rearing water sampling

Rearing water samples were collected in triplicate from each pond or cage from May to June 2022. At each farm, the water body was divided into six approximately equal subareas, three of which were randomly selected for sampling. From each selected location, 1 L of water was collected at approximately 20 cm depth using a sterile grab sampler and transferred into sterile containers.

Water temperature (WT), dissolved oxygen (DO), pH, salinity, and total dissolved solids (TDS) were measured immediately after sampling. DO, temperature, and salinity were recorded using a multiparameter handheld meter (model HI-98194, Hanna Instruments, Woonsocket, RI, USA; measurement ranges 0.00–50.00 mg L^-1^, 0.00–70.00 PSU, and 0–400.0 g L^-1^, respectively). The pH was measured using a portable pH meter (SevenCompact S210, Mettler Toledo, Columbus, OH, USA) calibrated daily with standard buffers. Nitrate, total ammonia, and iron concentrations were determined using colorimetric test kits (Tetra GmbH, Herrenteich, Germany) and a benchtop spectrophotometer (C-7000V, Peak Instruments, Shanghai, China), with detection limits of 100 mg L^-1^, 5.0 mg L^-1^, and 1.0 mg L^-1^, respectively.

Aliquots for physicochemical analyses were stored at 4 °C and processed at the Center of Excellence in Fish Infectious Diseases, Chulalongkorn University. Samples designated for DNA extraction were transported to the laboratory on ice, stored at −20 °C, and processed within 48–72 h of collection.

#### Fish gill sampling

Fish were euthanized by immersion in an ice–water bath as described previously. Gill arches were excised from each fish using sterile dissection instruments. Instruments were either dedicated to individual fish or thoroughly rinsed and disinfected with 70% ethanol between dissections to minimize cross-contamination. Gill arches obtained from fish at the same location were pooled to form a single composite sample.

To reduce bias associated with within-gill spatial heterogeneity, whole gills were collected following the methodology described by Clinton et al. [34]. The composite gill samples were placed in sterile tubes, transported on ice, and stored at −80 °C until DNA extraction and sequencing.

### Physicochemical measurements of water

The following water physicochemical variables were measured at each sampling location: ambient air temperature, WT, dissolved oxygen (DO), pH, salinity, conductivity, TDS, nitrate, total ammonia, and iron. The instrument models, manufacturers, and measurement ranges are detailed in the Methods section and summarized in Supplementary Table S1. For each province and farming system, mean ± standard deviation (SD) values were calculated to characterize the environmental gradients across the study sites.

### DNA extraction and preparation of libraries

Genomic DNA was extracted from water and composite gill samples using the DNeasy PowerWater Kit and DNeasy PowerFecal Pro DNA Kit (Qiagen, Hilden, Germany; catalog nos. 14900-100-NF and 51804, respectively), following the manufacturer’s instructions with minor adjustments. Water samples (1 L) were filtered through 0.22 μm cellulose nitrate membrane filters (MilliporeSigma, Burlington, MA, USA) prior to extraction. Filters and gill tissue (approximately 200 mg) were homogenized by vortexing with bead-beating tubes before lysis.

The DNA concentration and purity were assessed using a NanoDrop ND-1000 spectrophotometer (NanoDrop Technologies Inc., Wilmington, DE, USA), with typical yields of 5–25 ng μL^-1^ and A260/280 ratios between 1.8 and 2.0, consistent with high-quality fungal DNA.

The ITS1–ITS2 region of the fungal rRNA operon was amplified using the primer pair ITS1F (5ʹ-CTTGGTCATTTAGAGGAAGTAA-3ʹ) and ITS2 (5ʹ-GCTGCGTTCTTCATCGATGC-3ʹ), which targets the ITS1 region between the 18S and 5.8S rRNA genes. The polymerase chain reaction (PCR) mixture (25 μL) contained 10 ng of template DNA, 1× SparQ HiFi PCR Master Mix (QuantaBio, Beverly, MA, USA; catalog no. 95192-050), and 0.4 μM of each primer. No additional DNA polymerase was added because the enzyme was supplied in the master mix.

The thermal cycling conditions were as follows: initial denaturation at 98 °C for 2 min, followed by 30 cycles of 98 °C for 20 s, 60 °C for 30 s, and 72 °C for 1 min, and a final extension at 72 °C for 1 min.

Amplicons were purified using sparQ PureMag Beads (QuantaBio) and indexed with Nextera XT index primers in 50-μL reactions via 8–10 additional PCR cycles under the same cycling conditions. Indexed libraries were cleaned, quantified, and pooled at an equimolar ratio and then diluted to a final loading concentration of 4 pM with an internal control of 15% PhiX spike-in.

Libraries were sequenced on an Illumina MiSeq platform (Illumina, San Diego, CA, USA) using a MiSeq Reagent Kit v3 (Illumina, Inc., California, USA) (2 × 300 bp, 600 cycles) configuration at the Omics Sciences and Bioinformatics Center, Chulalongkorn University, Thailand. The number of raw read pairs in the 42 ITS libraries ranged from 3,205 to 594,695 per sample (median, 48,056). The number of denoized, nonchimeric reads per sample ranged from 2,097 to 342,212 (median, 34,543), corresponding to a median retention of 72.3% after quality filtering, merging, and chimera removal (Supplementary Table S2).

For each extraction and sequencing batch, extraction blanks and no-template PCR controls were included during library preparation. These negative controls did not yield visible ITS amplicons or retained amplicon sequence variants (ASVs) after denoising in QIIME 2, indicating negligible reagent or airborne contamination.

### Sequence quality control and classification of taxa

The raw demultiplexed reads were processed using QIIME 2 (version 2020.8). Quality filtering, denoising, paired-end read merging, and chimera removal were performed using the q2-dada2 plugin, with truncation lengths of 240 bp for forward reads and 200 bp for reverse reads based on inspection of Phred quality profiles and a minimum Phred score threshold of 20. ASVs were inferred per run and merged into a single feature table.

Taxonomic classification of ASVs was conducted using a naïve Bayes classifier trained on the UNITE reference database (version 8.3, dynamic release; ITS region, https://unite.ut.ee) via the q2-feature-classifier plugin. To reduce potential sequencing artifacts, extremely rare ASVs were removed prior to diversity analyses by excluding features with a total abundance of less than 10 reads across all samples.

Representative ASV sequences were aligned using MAFFT (version 7.471, https://mafft.cbrc.jp/alignment/ software), and a phylogenetic tree was constructed using FastTree (version 2.1.10, http://www.microbesonline. org/fasttree) under a general time-reversible model. Per sample DADA2 denoising statistics (input, filtered, merged, and nonchimeric read counts) were used to summarize sequencing depth and retention across samples; these metrics are reported in Supplementary Table S2.

### Diversity and statistical analyses

All diversity and community analyses were conducted on the rarefied ASV table subsampled without replacement to 11,979 sequences per sample. This rarefaction depth was selected on the basis of rarefaction curves for observed features, Shannon diversity, and Faith’s PD, and 34 of 42 libraries (81.0%) were retained, excluding eight low-depth samples.

Alpha diversity indices (observed ASVs, Shannon index, and Faith’s PD) were calculated in QIIME 2 and imported into R (version 4.0.3; R Core Team, 2020) for statistical analysis. Two-way analysis of variance (ANOVA) was used to assess the effects of sampling source (water vs gills), province, and farming style (open vs closed) when assumptions were met. The normality of the residuals was evaluated using the Shapiro–Wilk test, and homogeneity of variance was assessed using Levene’s test. When ANOVA assumptions were violated, nonparametric Kruskal–Wallis tests with pairwise Wilcoxon rank–sum post hoc comparisons were applied. Means and confidence intervals were visualized using interaction plots and boxplots.

For beta diversity analysis, the rarefied ASV table was converted to relative abundances before calculating Bray–Curtis and Jaccard dissimilarity matrixes. These distance matrixes were used for nonmetric multidimensional scaling (NMDS) ordination and permutational multivariate ANOVA (PERMANOVA). NMDS was performed using the metaMDS function in the vegan package (version 2.5-7) with multiple random starts (set.seed = 9999), and ordinations were visualized using base R plotting functions and ggplot2 (version 3.3.3). Where appropriate, convex hulls (ordihull) were used to illustrate group clustering.

PERMANOVA was conducted using the adonis2 function (vegan; 9,999 permutations) on Bray–Curtis distance matrixes to test for differences in community composition among sampling sources, provinces, and farming styles, reporting pseudo-F, R², and p values. Multivariate homogeneity of group dispersion was evaluated using the betadisper function (vegan) applied to Bray–Curtis distance matrixes for sampling source, province, and farming style. Tests were based on distances to group centroids in principal coordinate space, with significance assessed by F tests. PERMANOVA results were interpreted in the context of these dispersion tests.

The taxonomic composition was summarized at the genus and family levels by aggregating ASV counts from the rarefied table. Genus level bar plots were generated to visualize dominant taxa, and family-level heatmaps were produced using the heatplus and vegan packages in R. The relative abundances of key opportunistic or potentially pathogenic genera (e.g., *Cladosporium*, *Phoma*, *Aspergillus*, *Fusarium*, *Candida*, and *Rhodotorula*) were summarized descriptively and highlighted in the plots.

Canonical correlation analysis was used to explore multivariate associations between mycobiome diversity and environmental conditions in gill and rearing water samples. The response set for each habitat comprised Shannon diversity and Faith’s PD, and the predictor set comprised ambient temperature, WT, dissolved oxygen, pH, salinity, conductivity, TDS, nitrate, total ammonia, and iron concentrations. All environmental variables were z-standardized (mean = 0, SD = 1) before analysis.

Multicollinearity among predictors was assessed by calculating variance inflation factors (VIFs) from a subset of samples with complete physicochemical data. As expected, the three ionic strength variables (salinity, conductivity, and TDS) and nitrate were strongly collinear (VIFs > 10 and, in some cases, > 50), whereas the remaining predictors showed only moderate collinearity (VIFs < 10). Given the exploratory nature of the canonical correlation analyses and the conceptual relevance of these gradients, the full set of predictors was retained, and canonical coefficients of ionic strength variables were interpreted as representing a correlated environmental block rather than independent effects. Canonical correlations and their significance were evaluated using Wilks’ λ and approximate F tests at α = 0.05.

### Quality assurance and biosafety

All laboratory work involving fungal DNA extraction, PCR, and amplicon library preparation was conducted in a biosafety level-2 laboratory using certified biological safety cabinets. To minimize contamination, aerosol-resistant filter tips, sterile consumables, and routine surface decontamination with 70% ethanol and appropriate disinfectants were used.

Extraction blanks and no-template PCR controls were processed alongside samples throughout DNA extraction, amplification, and sequencing, and denoising did not yield visible amplicons or retained ASVs. Biological waste, including fish tissues, used filters, media, and residual nucleic acids, was autoclaved or chemically disinfected prior to disposal in accordance with institutional biosafety guidelines.

## RESULTS

### Sequence output and rarefaction

Illumina MiSeq ITS amplicon sequencing generated libraries for 42 samples (rearing water and gill microbiomes across open-cage and closed-pond systems). The number of raw reads per sample ranged from 3,205 to 594,695 (median, 48,056 reads). After DADA2 quality filtering, denoising, merging, and chimera removal, a median of 34,543 nonchimeric reads per sample were retained (range, 2,097–342,212), corresponding to a median retention of 72.3% of the input reads (range, 7.1–90.4%; Supplementary Table S2).

**Figure 2:**
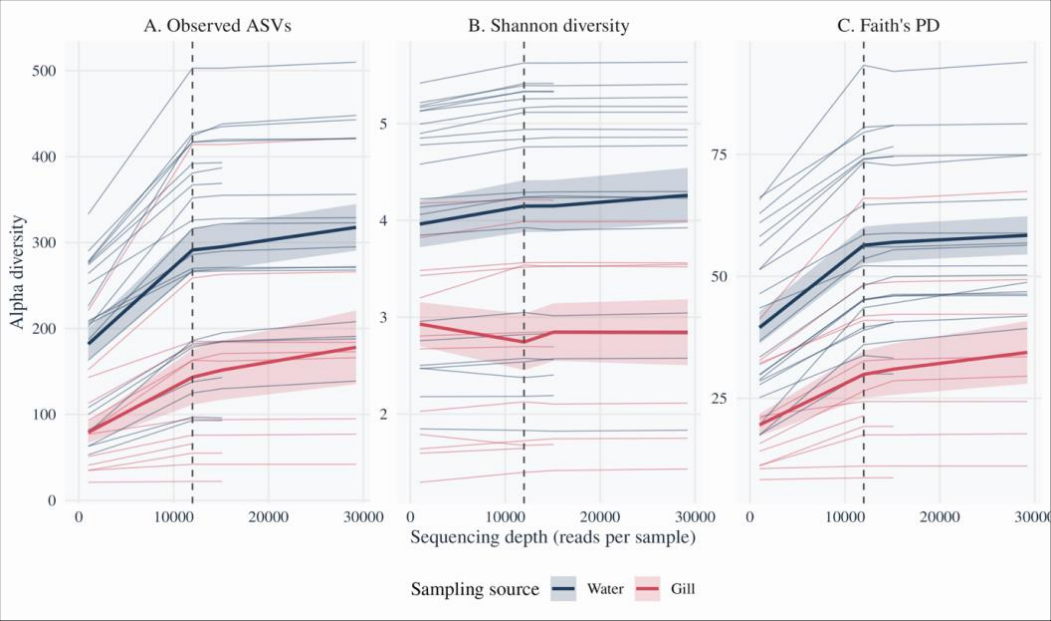
Rarefaction curves of fungal alpha diversity in rearing water and gills. Rarefaction curves showing the relationship between sequencing depth and fungal alpha diversity for all ITS libraries (n = 42). Each line represents one sample, and the curves are colored according to the sampling source (blue = rearing water, orange = gills). The vertical dashed line indicates the rarefaction depth of 11,979 reads per sample, at which 34 of 42 libraries were retained for downstream diversity analyses. Panels show (A) observed amplicon sequence variants (ASVs), (B) Shannon diversity, and (C) Faith’s phylogenetic diversity (PD) as functions of sequencing depth. Shaded ribbons represent the means ± standard errors across samples within each sampling source.

The rarefaction curves for observed ASV richness, Shannon diversity, and Faith’s PD flattened at approximately 10,000–12,000 sequences per sample (Figures 2A–C), indicating that sequencing depth was generally sufficient to capture most fungal richness. The denoized feature table was rarefied to 11,979 sequences per sample to standardize sampling effort. This depth retained 34 of 42 libraries (81.0%) and excluded eight low-depth libraries (six gill and two rearing water samples) from downstream alpha diversity and beta diversity analyses.

### Alpha diversity

**Figure 3:**
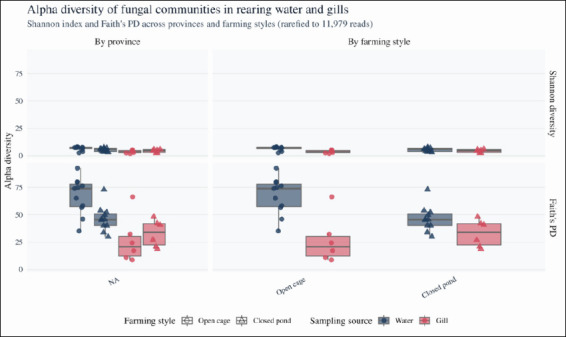
Alpha diversity of fungal communities in rearing water and gills across provinces and farming styles. Boxplots showing Shannon diversity and Faith’s phylogenetic diversity (PD) for rearing water and gill mycobiomes across provinces and farming styles. Boxes indicate the interquartile range (IQR), horizontal lines represent medians, whiskers extend to 1.5 × IQR, and points denote individual samples (rearing water: n = 23; gills: n = 18). Colors indicate the sampling source (blue = rearing water, orange = gills), and shapes represent farming style (circles = open river cages, triangles = closed earthen ponds). Differences in Shannon diversity and Faith’s PD were tested using two-way analysis of variance with sampling source, province, and farming style as fixed factors.

Fungal alpha diversity was assessed using the Shannon diversity index (Shannon ID), which incorporates both richness and evenness, and Faith’s PD, which summarizes the total branch length of the phylogenetic tree. Across the rarefied dataset, rearing water communities consistently presented greater diversity than gill communities. The mean Shannon diversity was 6.11 ± 1.77 in water (n = 21) and 3.96 ± 1.46 in gills (n = 12), whereas mean Faith’s PD was 57.87 ± 17.30 in water and 29.83 ± 16.94 in gills (Figure 3).

Two-way ANOVA was used to test the effects of sampling source (rearing water vs gills), province, and farming style (open-cage vs closed-pond) on Shannon ID and Faith’s PD (Table 2). The model residuals satisfied normality and homogeneity assumptions (Shapiro–Wilk and Levene’s tests; data not shown); therefore, ANOVA results were retained. Sampling source had a significant effect on both Shannon ID (F_1,27_ = 8.96, p = 0.0058) and Faith’s PD (F_1,27_ = 15.05, p = 6.08 × 10^-4^), whereas neither province nor farming style significantly influenced either metric (Table 2). When assumptions were not met in additional exploratory contrasts, alpha diversity indices were analyzed using the Kruskal–Wallis test with pairwise Wilcoxon rank–sum post hoc comparisons. Overall, these results indicate that fungal species and PD are structured primarily by habitat (water vs gills), with no detectable differences in alpha diversity among provinces or between open and closed systems at the chosen sequencing depth.

**Table 2:** Two-way analysis of variance revealing the influence of sampling source, sampling location (province), and farming style on fungal diversity based on Shannon index and Faith’s phylogenetic diversity (PD).

Diversity metrics	Variables	Df	Sum Sq	F value	Pr (>F)
Shannon ID	Sampling source	1	22.35	8.96	0.00584
	Province	4	22.51	2.26	0.08935
	Farming style	1	0.65	0.26	0.61390
Faith’s PD	Sampling source	1	4648.38	15.05	6.08E-04
	Province	4	282.62	0.23	0.91983
	Farming style	1	508.24	1.65	0.21043

### Beta diversity

**Figure 4:**
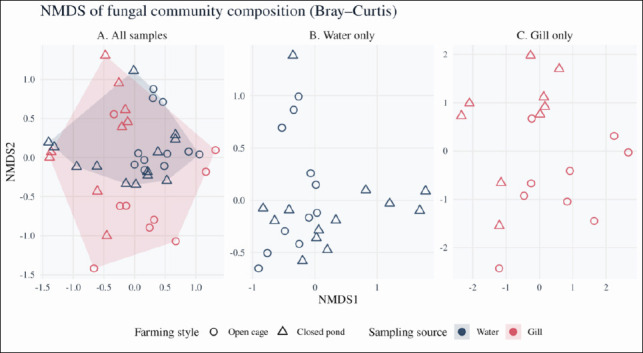
Nonmetric multidimensional scaling (NMDS) analysis of fungal community composition. NMDS ordinations based on Bray–Curtis dissimilarities of relative ITS ASV abundances. (A) Combined dataset including all samples (n = 42). (B) Rearing water samples only (n = 23). (C) Gill samples only (n = 18). Points are colored according to sampling source (blue = rearing water, orange = gills) and shaped according to farming style (circles = open river cages, triangles = closed earthen ponds). Ellipses or convex hulls indicate the dispersion of groups within the ordination space. The global NMDS solution for all samples (A) showed a stress value of 0.216. Permutational multivariate analysis of variance (PERMANOVA) of Bray–Curtis distances (999 permutations) revealed significant effects of sampling source (R² = 0.055, p < 0.001), province (R² = 0.193, p < 0.001), and farming style (R² = 0.061, p < 0.001) on fungal community composition.

Beta diversity patterns were evaluated using Bray–Curtis dissimilarities derived from relative abundances of ASVs and visualized using NMDS. The global NMDS ordination (stress = 0.216) revealed clear compositional separation between rearing water and gill-associated fungal communities (Figures 4A and 4B). PERMANOVA indicated that sampling source, province, and farming style each explained a significant portion of the variation in community structure (source: pseudo-F = 2.81, R² = 0.055, p = 0.0001; province: pseudo-F = 2.49, R² = 0.193, p = 0.0001; farming style: pseudo-F = 3.16, R² = 0.061, p = 0.0001).

Separate NMDS analyses of water and gill samples indicated that both province and farming style significantly affected beta diversity within each habitat. For rearing water, PERMANOVA indicated significant effects of province (pseudo-F = 3.10, R² = 0.357, p = 0.0001) and farming style (pseudo-F = 2.53, R² = 0.113, p = 0.0005). For gills, province (pseudo-F = 2.01, R² = 0.360, p = 0.0001) and farming style (pseudo-F = 2.53, R² = 0.113, p = 0.0005) significantly influenced community composition. Dotted convex hulls in the ordination highlight distinct clustering patterns between water and gill samples from open versus closed systems.

Multivariate homogeneity of dispersion (betadisper) tests indicated no significant differences in dispersion among provinces or between farming systems (F ≤ 0.73, p ≥ 0.44). In contrast, gill-associated communities displayed slightly greater dispersion than rearing water communities (F_1,40_ ≈ 4.69, p ≈ 0.036), suggesting that the significant PERMANOVA effect for sampling source reflects both differences in group centroids and modest within-group variability. The effect size for sampling source remained small (R² ≈ 0.05), which is consistent with a minor but detectable shift in fungal community composition between gill and water habitats.

### Mycobiome composition and abundance

**Figure 5:**
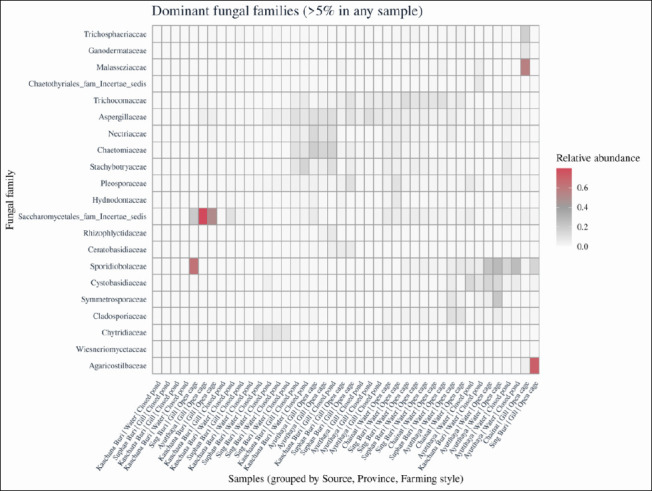
Heatmap of dominant fungal families in rearing water and gill samples. Heatmap showing the relative abundance of fungal families contributing >5% of total reads in at least one sample. Columns represent individual samples grouped by sampling source, province, and farming style, and rows represent fungal families. Color intensity from light to dark red indicates increasing relative abundance. Multivariate homogeneity of group dispersion was evaluated using the betadisper function in the vegan package (Bray–Curtis distances) for sampling source, province, and farming style prior to interpreting permutational multivariate analysis of variance results.

Across all samples, 21 fungal families each accounted for >5% of total reads in at least one library, although five of these families could not be fully resolved to a named family based on ITS sequences. A heatmap of family-level relative abundances (Figure 5) revealed significant differences between water and gill samples, with clear clustering by sampling source and, to a lesser extent, by province and farming style.

**Figure 6:**
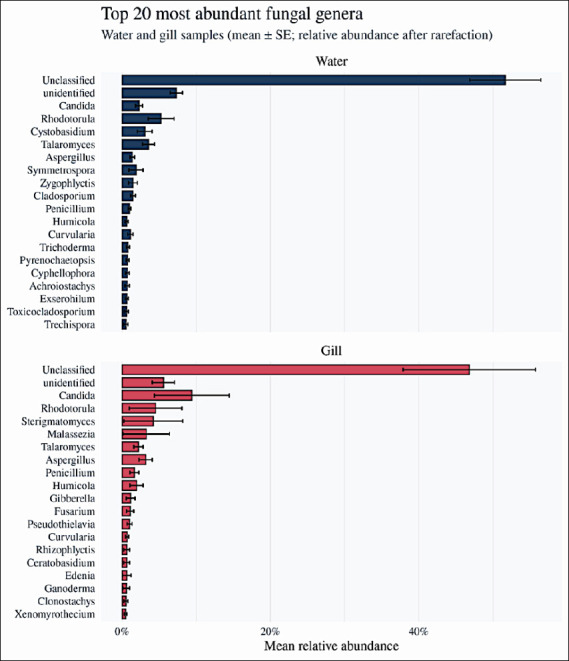
Top 20 most abundant fungal genera in rearing water and gill samples. Bar plots illustrating the 20 most abundant fungal genera in rearing water (n = 23) and gill samples (n = 18) based on relative read abundance after rarefaction. Bars represent mean relative abundance across samples within each group, and error bars indicate ±1 standard error. The detected genera include both predominantly saprophytic taxa and genera with recognized pathogenic or opportunistic potential in fish and humans, including *Candida*, *Fusarium*, *Aspergillus*, *Rhodotorula*, and *Cladosporium*.

At the genus level, the top 20 most abundant taxa in gill and rearing water communities included several genera with recognized pathogenic potential in tilapia or other fish (Figure 6), notably *Candida*, *Fusarium*, *Aspergillus*, and *Rhodotorula*. *Candida* and *Fusarium* were more prominent in gill samples, whereas Cladosporium and *Rhodotorula* were enriched in rearing water. To evaluate how the combined relative abundance of these four genera varied with production system, we fitted a two-way ANOVA with sampling source, province, and farming style as fixed factors. The model indicated significant effects of sampling source (F = 5.29, p = 0.0217), province (F = 5.40, p = 2.67 × 10^-4^), and farming style (F = 22.38, p = 2.62 × 10^-6^ Table 3), confirming that both spatial location and rearing system influence the distribution of potentially pathogenic fungi.

**Table 3:** Two-way analysis of variance summarizing the potential effects of sampling source, sampling location (province), and farming style on the relative abundance of four fungal tilapia pathogens (n = 34 samples after rarefaction). *p < 0.05, p < 0.01.

Variables	Sum Sq	Df	F value	Pr (>F)
Sampling source	118905	1	5.2875	0.021719
Province	486150	4	5.4046	0.000267
Farming style	503167	1	22.3751	2.62E-06

Sum Sq: Sum of squares; Df: Degrees of freedom; F value: F-statistic; Pr (>F): Probability value (p-value) associated with the F-statistic.

**Figure 7:**
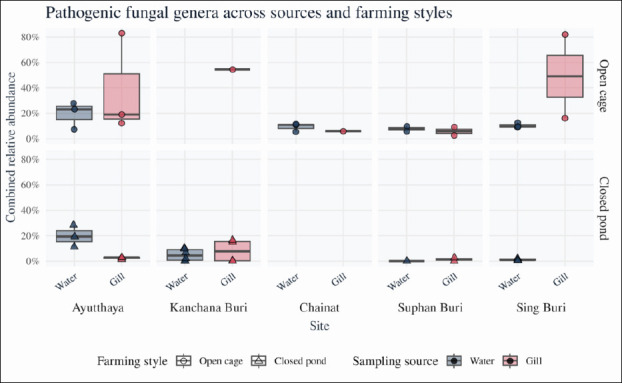
Relative abundance of potentially pathogenic fungal genera across sampling sources and farming styles. Boxplots showing the combined relative abundance of four tilapia-associated fungal genera (*Aspergillus*, *Fusarium*, *Candida*, and *Rhodotorula*) in rearing water and gill samples from open river cages and earthen ponds. The combined abundance was calculated as the sum of the relative abundances of these genera divided by the total ITS read count per sample. Boxes indicate the interquartile range, horizontal lines represent medians, whiskers extend to 1.5 × IQR, and points denote individual samples (rearing water: n = 23; gills: n = 18). Differences in combined pathogen abundance were assessed using two-way analysis of variance with sampling source, province, and farming style as fixed factors.

The relative abundances of six opportunistic and potentially pathogenic genera (*Aspergillus*, *Fusarium*, *Cladosporium*, *Phoma*, *Candida*, and *Rhodotorula*) were assessed across habitats. Collectively, these taxa accounted for a median of 7.9% of total ITS reads in gill libraries (range ~0.1–82.9%) and 10.9% in rearing water libraries (range ~0.0–30.4%). At the genus level, *Aspergillus* typically represented 1%–2% of reads (median 1.4% in gills, 0.9% in water), with maxima of 9.6% and 5.9%, respectively. *Fusarium* was generally less abundant (median 0.0% in gills, 0.1% in water) but reached 7.3% and 2.6% in some gill and water samples, respectively. *Cladosporium* was more prominent in water (median 1.0%, up to 6.6%) than in gills (median 0.0%, up to 5.3%). These patterns in the combined relative abundance of pathogenic genera across habitats and farming systems are illustrated in Figure 7, and Table 3 summarizes the corresponding two-way ANOVA results.

Yeast genera were prevalent across both water and gill habitats. *Candida* presented a median relative abundance of 1.1% in gills and 1.6% in water, but it was dominant in some individual gill samples, accounting for up to ~80% of reads. *Rhodotorula* presented a median abundance of 0.0% in gills and 1.3% in water, with peak values of ~63% and ~27%, respectively. *Phoma* was detected only at trace levels (≤0.1% of reads) in a minority of water samples and was absent from the gills. In the absence of established quantitative thresholds for fungal dysbiosis in tilapia, these abundances are interpreted as a baseline yet potentially loaded state in clinically healthy fish, where a high representation of opportunistic genera may serve as an early warning indicator under additional environmental or husbandry stressors.

### Water physicochemical properties

Physicochemical conditions typical of warm-water freshwater aquaculture in lowland Thailand are shown in Supplementary Table S1. Atmospheric temperature and WT ranged from 32.8°C to 35.6°C and 29.6°C to 33.4°C, respectively, with most ponds having temperatures between 31°C and 33°C. Dissolved oxygen levels were generally within ranges suitable for tilapia culture (5.7–8.1 mg/L^-1^). Water pH ranged from moderately acidic to nearly neutral (5.46–7.67), with slightly lower values in some closed-pond systems and more neutral conditions in several open cages. Salinity, conductivity, and total dissolved solids showed parallel patterns, with salinity of approximately 63–327 mg L^-1^, conductivity of approximately 134–448 μS cm^-1^, and TDS of approximately 93–327 mg L^-1^, and these tended to be higher in certain closed-pond setups. Overall, inorganic nitrogen levels were low to moderate: nitrate ranged from approximately 0.001 to 2.50 mg L^-1^, whereas total ammonia ranged from approximately 1.23 to 4.53 mg L^-1^, with the highest values observed in Sing Buri ponds. Dissolved iron concentrations were generally low (0.01–0.53 mg L^-1^). These data provide the physicochemical context for the rearing water and gill mycobiomes analyzed in this study and ensure that the manuscript is self-contained with respect to environmental conditions.

### Associations between environmental variables and fungal diversity

To investigate how local environmental conditions may influence fungal diversity, correlations were examined between mycobiome diversity metrics (Faith’s PD and Shannon ID) and measured physicochemical variables in gill and rearing water samples (Table 4). Overall, correlations ranged from weak to moderate. WT showed minimal associations with either diversity metric (r ≤ 0.37), whereas ambient air temperature was moderately correlated with gill Shannon diversity (r ≈ 0.64).

**Table 4:** Correlation matrix of mycobiome diversity (gill and water samples) and environmental variables (n = 34 samples). Bold correlations are statistically significant at p < 0.05.

Parameter	Faith’s PD (Gill)	Faith’s PD (Water)	Shannon ID (Gill)	Shannon ID (Water)
Ambient temperature	0.212	0.435	0.637	0.292
Water temperature	−0.137	0.038	−0.370	0.033
Dissolved oxygen	−0.008	−0.112	−0.203	−0.355
pH	−0.770	0.436	−0.763	0.476
Salinity	0.268	−0.171	0.309	−0.134
Conductivity	0.329	−0.242	0.364	−0.198
Total dissolved solids	0.339	−0.236	0.398	−0.192
Nitrate	0.610	−0.364	0.702	−0.541
Total ammonia	−0.286	0.316	0.030	0.425
Iron	−0.211	0.347	−0.265	0.257

PD = Phylogenetic diversity.

Gill diversity was strongly related to pH and nitrate. Both Faith’s PD and Shannon ID were negatively associated with pH (r ≈ −0.76 to 0.77) and positively associated with nitrate (r ≈ 0.61–0.70), suggesting that slightly more acidic, nitrate-enriched conditions corresponded with richer and more phylogenetically diverse gill mycobiomes. In contrast, Shannon diversity in rearing water was negatively correlated with nitrate (r ≈ −0.54), indicating that elevated nitrate was associated with reduced taxonomic evenness in the water column. Ionic strength indicators (salinity, conductivity, and TDS) tended to correlate positively with gill diversity but were slightly negatively associated with water Shannon diversity. Total ammonia and iron were positively correlated with water diversity indices, particularly water Shannon ID (r ≈ 0.43 for total ammonia; r ≈ 0.26 for iron).

Canonical correlation analysis was used to summarize multivariate relationships between mycobiome diversity and water chemistry in gill and rearing water datasets. For each habitat, Shannon ID and Faith’s PD were treated as response variables, and 10 physicochemical parameters were treated as predictors. Environmental variables were z-standardized and screened for collinearity before analysis. For gill samples, neither canonical dimension was statistically significant, indicating no strong multivariate association between diversity and environmental variables. For rearing water samples, the first canonical dimension showed a strong and statistically significant association between diversity indices and water chemistry (canonical correlation = 0.85, p = 0.017), whereas the second dimension was not significant (p = 0.078). Supplementary Tables S3 and S4 present summary statistics for the canonical correlation analyses, whereas Supplementary Tables S5 and S6 provide complete canonical statistics and standardized coefficients.

## DISCUSSION

### Study context and objectives

Over the past few decades, microbiome-based approaches have emerged as promising tools to improve aquaculture productivity, health, and sustainability, particularly in low- and middle-income countries where farmed fish are critical for food security and livelihoods [1, 2, 7, 19]. However, most studies have focused on bacterial communities, and relatively little is known about how different rearing systems shape the diversity and composition of fungal communities in rearing water and on fish mucosal surfaces [13, 18, 19]. This study aims to fill this knowledge gap by characterizing the mycobiomes of rearing water and the gills of clinically healthy red tilapia (*Oreochromis* spp. hybrids) raised in earthen ponds and open river cages in Central Thailand. This study further links fungal diversity patterns to local water quality conditions within a broader One Health framework.

### Hypotheses

Based on previous research on fish microbiomes and differences between intensive and semi-intensive production systems [7, 15, 20, 32, 35-37], we created three hypotheses. First, we predicted that fungal alpha and PD (Shannon index and Faith’s PD) would be greater in rearing water than in gills, suggesting that the water column is a heterogeneous environmental reservoir for fungal propagules and that the gills are a more selective mucosal interface [17, 19, 20, 37, 38]. Second, we hypothesized that fungal community composition (beta diversity) would vary between open river cages and closed earthen ponds, as well as among provinces, in accordance with differences in water exchange, nutrient loading, and local environmental conditions [13, 23, 24, 26, 36, 39]. Third, we anticipate that opportunistic and potentially pathogenic genera, such as *Aspergillus*, *Fusarium*, *Candida*, *Rhodotorula*, and *Cladosporium*, would be detectable in both water and gill samples, with relatively enriched mucosa-associated taxa on gill surfaces [7, 13, 29, 40–45].

### Evidence for habitat-driven differences in alpha diversity

Our diversity analyses supported the first hypothesis: rearing water communities consistently exhibited greater fungal diversity than gill communities, in both Shannon diversity and Faith’s PD. This pattern aligns with broader teleost microbiome studies showing that host-associated communities differ from those in the surrounding water, with gill and skin microbiota forming structured assemblages that only partially overlap with those in planktonic reservoirs [15, 17, 25, 27, 35, 36]. The water column functions as an environmental reservoir of fungal propagules derived from catchment inflows, feeds, and infrastructure, whereas gill epithelia are lined with mucus, lectins, and other immune factors that impose strong ecological filtering on colonizing microbes [20, 46–48]. In this context, the greater diversity observed in rearing water and the subset-like composition of gill communities are consistent with a model in which the gills selectively recruit or retain only a fraction of the available fungal taxa.

In contrast, alpha diversity metrics did not differ significantly among provinces or farming styles, suggesting that the host compartment (water vs gill) exerts a stronger influence on local fungal richness and phylogenetic breadth than broader geographic or management gradients at the scales and sample sizes examined.

### Compositional structuring and drivers of beta diversity

Nevertheless, the beta diversity analyses revealed clear compositional structure. PERMANOVA of Bray–Curtis dissimilarities revealed that sampling source (gill vs rearing water), province, and farming style together explained a substantial fraction of the variance in community composition, with province and rearing system accounting for approximately 25%–35% of variation when water and gill communities were analyzed separately. This finding is consistent with previous work demonstrating that primary drivers of microbial turnover in aquaculture systems are site-level environmental conditions and management (e.g., nutrient loading, hydrology, salinity, and water exchange) [11, 13, 23, 25, 26, 36, 39, 49]. In practical terms, our results suggest that interventions targeting water quality and farm management are likely to have stronger effects on fungal assemblages than measures aimed solely at individual fish and that location- and practice-specific strategies may be required to effectively manage mycobiome composition.

### Environmental contrasts between ponds and cages

Environmental differences between closed earthen ponds and open river cages likely contributed to these compositional patterns. Closed ponds typically have reduced water exchange, greater organic matter retention, and greater nutrient accumulation, which can promote fungal proliferation and niche diversification [26, 39, 49]. In contrast, open cages are more strongly coupled to river hydrology, with continuous water turnover that can dilute metabolites and reduce the build-up of organic substrates, but also expose fish to catchment-scale contaminants and propagule fluxes [30, 31, 49]. In the present study, water quality parameters across farms fell within ranges compatible with tilapia culture [9, 10], yet subtle differences in pH, salinity, ionic strength, and inorganic nitrogen were evident between some pond and cage sites. These gradients are known to influence fungal diversity and community composition in aquatic systems [13, 23, 24, 50, 51] and likely underpin part of the beta diversity structure observed among provinces and farming styles.

### Dominant taxa and ecological interpretation

Our mycobiome profiles were dominated by yeasts and filamentous fungi that are frequently reported in freshwater and aquaculture environments in terms of taxonomic composition. *Rhodotorula* spp.’s prominence in rearing water is consistent with their ecology, as saprophytic yeasts adapt to organic-rich and sometimes stressful aquatic niches [13, 52, 53]. Marine and freshwater *Rhodotorula* strains are being explored as alternative lipid and docosahexaenoic acid sources for fish larvae [52]. Several isolates have been shown to efficiently assimilate dissolved organic carbon and tolerate metals, making them candidates for bioremediation [52, 54]. *Rhodotorula mucilaginosa* and related species have emerged as opportunistic pathogens in humans and animals, particularly in immunocompromised hosts and in nosocomial settings [53, 55, 56]. Therefore, detection of Rhodotorula on gills and in rearing water suggests a dual ecological role: contributions to nutrient cycling and biofilm formation, alongside potential opportunistic risk under permissive conditions.

### Opportunistic yeasts and filamentous fungi

*Candida* spp. were also widespread in water and gill samples. *Candida* is a typical mucosal commensal in many vertebrates but can cause invasive disease when epithelial barriers are breached or immunity is impaired [14, 45]. *Candida* spp. have been implicated in mycotic lesions of skin and gills and in systemic infections in fish, often in association with stress or co-infection [45, 57]. Candida occasionally dominated individual gill libraries in our dataset, reaching very high relative abundances despite the absence of overt disease. This pattern supports the idea of a “loaded but compensated” state in clinically healthy tilapia, in which high representation of opportunistic yeasts may serve as an early warning signal when combined with additional environmental or husbandry stressors rather than indicating dysbiosis on its own.

Filamentous genera such as *Cladosporium*, *Aspergillus*, and *Fusarium* are also common features of the water–gill mycobiome. *Cladosporium* is frequently recovered from aquatic environments and is recognized as both a spoilage organism and a potential pathogen in immunocompromised hosts [40, 54]. *Aspergillus* species are well-established opportunistic pathogens of both fish and humans [29, 58-61] and have been reported as dominant contaminants in aquafeeds and potable water distribution systems [28, 41, 42, 62]. Similarly, *F. oxysporum* and related species can cause skin and subcutaneous mycoses in tilapia and other fish, often in co-infection with bacterial pathogens such as *A. hydrophila* [44, 61]. In the present study, *Aspergillus* and *Fusarium* were more prominent in gill samples than in rearing water for some farms, whereas Cladosporium tended to be enriched in water. *Phoma* is an environmentally ubiquitous genus that includes phytopathogenic and opportunistic species with recognized clinical relevance, although detected only at very low levels and only in Kanchanaburi [63]. The combined relative abundance of these opportunistic and potentially pathogenic genera (*Aspergillus*, *Fusarium*, *Cladosporium*, *Phoma*, *Candida*, and *Rhodotorula*) accounted for approximately 8%–11% of the reads on average but occasionally dominated individual libraries. Given the absence of clinical disease and the lack of quantitative thresholds for fungal dysbiosis in tilapia, we interpret these profiles as baseline but potentially “loaded” community states that could tip toward disease under unfavorable conditions.

### Links between water chemistry and fungal diversity

Correlations between fungal diversity indices and water physicochemical variables further emphasize the role of environmental filtering. In gill samples, Shannon diversity and Faith’s PD were negatively associated with pH but positively associated with nitrate, suggesting that slightly more acidic and nitrate-enriched conditions were associated with richer and more phylogenetically diverse mycobiomes. In contrast, nitrate concentration in rearing water was negatively related to Shannon diversity, indicating reduced evenness at relatively high nitrate concentrations. Ionic strength proxies (salinity, conductivity, and TDS) tended to correlate positively with gill diversity but weakly or negatively with water diversity. These patterns are broadly consistent with studies showing that nutrient enrichment and water chemistry can modulate fungal diversity in rivers, ponds, and coastal sediments [13, 23, 26, 50, 51]. Canonical correlation analyses, which simultaneously integrate multiple physicochemical variables, revealed a strong multivariate association between water chemistry and fungal diversity in rearing water but weaker or nonsignificant associations for gill communities, which is consistent with the idea that both environmental drivers and host-mediated processes shape gill mycobiomes [15, 20, 35, 36].

### Mechanistic interpretation and future research needs

Taken together, our observations support a simple mechanistic model for these tilapia farms: rearing water functions as an environmental reservoir and mixing point for fungal propagules originating from catchment inflows, aquafeeds, and infrastructure, whereas the gills serve as a selective mucosal interface where only a subset of fungi successfully attach, persist, and occasionally invade [19, 37, 64]. Genera such as *Candida* and *Rhodotorula* are located at the intersection of these niches, reflecting both organic enrichment and sustained exposure of mucosal surfaces. Longitudinal studies integrating functional assays (e.g., mycotoxin production, enzymatic activities, and biofilm formation), antifungal susceptibility testing, and host health metrics with mycobiome profiling are needed to determine whether particular community configurations are benign signatures of intensive production or early indicators of heightened disease risk [7, 26, 41].

### One Health relevance and practical implications

From a health standpoint, the fungal communities described here are linked to the aquatic environment, farmed fish, and human populations. Several dominant or recurrent genera in our dataset, including *Aspergillus*, *Fusarium*, *Candida*, *Rhodotorula*, *Cladosporium*, and *Phoma*, are recognized as opportunists or emerging pathogens in humans and animals [14, 44, 53, 55-57, 61, 63]. Thus, tilapia aquaculture has the potential to act as a bridge between environmental reservoirs and fish disease, particularly where poor water quality, stocking stress, or coinfections compromise mucosal defenses [7, 29, 32, 40]. Moreover, farm workers can be repeatedly exposed to fungal spores and bioaerosols during routine activities such as feeding, net handling, and harvesting, and contaminated water or effluents may disseminate fungi to downstream users and ecosystems [28, 42, 62, 65].

These considerations underscore the value of incorporating fungal community profiling into aquaculture biosecurity plans and surveillance programs alongside existing bacterial and parasitic monitoring [8, 22, 40, 66]. By providing the first ITS rRNA–based characterization of fungal communities associated with red tilapia aquaculture systems in Central Thailand, this study establishes a regional baseline and a framework for future mycobiome-aware One Health surveillance in tropical freshwater aquaculture.

### Recommendations

Routine fungal monitoring in aquaculture systems is essential for detecting pathogenic and opportunistic fungi before they cause disease outbreaks, thereby safeguarding both fish health and food safety. Targeted biosecurity measures, including improved water management, effective disinfection protocols, and strict feed hygiene, can help reduce the persistence and spread of opportunistic fungi within the rearing environment. Risk assessment for farm workers handling tilapia should also be prioritized because occupational exposure to fungal spores and opportunistic pathogens may pose health risks, particularly for immunocompromised individuals.

Integrating fungal surveillance into broader One Health frameworks will facilitate the early detection of cross-sectoral threats by linking aquaculture, public health, and environmental monitoring to support safer and more sustainable aquaculture systems. These findings are consistent with the Food and Agriculture Organization and the World Organization for Animal Health One Health frameworks, which emphasize integrated surveillance of pathogens at the interface of aquatic animals, humans, and the environment in aquaculture systems.

Future studies should extend this work by increasing the number of farms and individual fish sampled per habitat and by covering a wider range of environmental conditions and production seasons. Larger and more balanced datasets will enable more robust multivariate analyses to test whether the associations observed between water chemistry, nutrient status, and mycobiome diversity across farming systems and seasons remain consistent and to assess the robustness of the ecological patterns suggested by the present study.

### Limitations of the study and future directions

This study has several limitations that should be considered when interpreting the results. First, sampling was restricted to ten farms across five provinces during a single production season (May–June 2022), which may not capture temporal or interannual variation in mycobiome structure or the full diversity of farming practices employed in Thai tilapia aquaculture. Second, the analyses relied solely on ITS rRNA amplicon sequencing. Histopathology, culture-based identification, and antifungal susceptibility testing were not performed; therefore, the pathogenic potential of the detected genera was inferred from taxonomic classification and existing literature rather than being directly confirmed in diseased tissue.

Third, all sampled fish were clinically healthy, and no quantitative host-level metrics such as growth performance, survival, immune parameters, or lesion scores were recorded. Consequently, the present study could not directly link specific mycobiome configurations with individual fish health outcomes. Finally, environmental characterization was limited to a subset of physicochemical parameters measured at the time of sampling. Additional potential drivers, including organic loading, feed inputs, farm-level antimicrobial use, and co-occurring bacterial or viral communities, were not quantified.

Accordingly, the mycobiome profiles described here should be considered a baseline reference for apparently healthy red tilapia (*Oreochromis* spp. hybrids) in Central Thailand rather than a definitive characterization of dysbiosis or disease states. Future investigations should incorporate longitudinal and multisession sampling combined with histopathology, fungal culture, antifungal susceptibility testing, and multiomic approaches such as shotgun metagenomics and metatranscriptomics. Integrating these approaches with host performance indicators, immune responses, and clinical observations would help elucidate causal relationships between environmental change, mycobiome dynamics, and mycotic disease risk in tilapia aquaculture. Such integrative research will support the development of mycobiome-informed surveillance and management strategies within a One Health framework for sustainable tropical aquaculture systems.

## CONCLUSION

This study provides the first comprehensive ITS rRNA–based characterization of fungal communities associated with rearing water and gill mucosa of clinically healthy red tilapia (*Oreochromis* spp. hybrids) cultured in open river cages and closed earthen ponds in Central Thailand. The results revealed clear habitat-driven patterns in fungal diversity and community composition. Rearing water communities consistently exhibited greater alpha and PD than gill-associated communities, supporting the hypothesis that the surrounding water functions as a heterogeneous environmental reservoir for fungal propagules, whereas the gill surface represents a selective mucosal interface that filters and retains only a subset of environmental taxa. Beta diversity analyses further demonstrated that fungal community composition differed significantly according to sampling source, province, and farming system, highlighting the role of environmental conditions and aquaculture management practices in structuring mycobiome composition.

Taxonomic analyses identified several dominant genera with recognized ecological or opportunistic pathogenic relevance in aquaculture environments, including *Candida*, *Fusarium*, *Aspergillus*, *Rhodotorula*, *Cladosporium*, and *Phoma*. Although these genera collectively represented a modest proportion of the overall fungal community, they occasionally dominated individual samples, even in the absence of overt disease. Correlation and canonical correlation analyses indicated that fungal diversity patterns were associated with physicochemical variables such as pH, nitrate concentration, and ionic strength, emphasizing the influence of environmental filtering on fungal community structure in aquaculture systems.

Overall, the findings highlight that fungal communities represent an integral yet often overlooked component of aquaculture microbiomes. By establishing baseline mycobiome patterns at the water–gill interface of farmed red tilapia in Central Thailand, this study contributes to a deeper ecological understanding of aquaculture microbial systems and underscores the importance of incorporating fungal surveillance into integrated aquatic animal health management and One Health monitoring frameworks.

## DATA AVAILABILITY

All the raw ITS rRNA amplicon reads generated in this study have been deposited in the National Center for Biotechnology Information Sequence Read Archive under BioProject accession number PRJNA1374232. The processed feature tables, taxonomic assignments, and environmental metadata used for the analyses are provided in Supplementary Tables S1–S6 and are also available upon reasonable request from the corresponding author.

## AUTHORS’ CONTRIBUTIONS

GBD: Conceptualization, experimental design, data analysis, manuscript drafting, and manuscript revision. MM: Data analysis, manuscript drafting, and manuscript revision. SC: Conceptualization, experimental design, data analysis, manuscript drafting, and manuscript revision. CR: Conceptualization, experimental design, performing the experiments, data analysis, manuscript drafting, and manuscript revision. All authors contributed to writing, reviewing, and editing the manuscript and approved the final version of the manuscript.
